# Formative Research for the Development and Implementation of a Smartphone Application to Report Breaches to the International Code of Marketing of Breast‐Milk Substitutes in Mexico

**DOI:** 10.1111/mcn.70014

**Published:** 2025-03-18

**Authors:** M. Unar‐Munguía, M. Ceballos‐Rasgado, P. J. Mota‐Castillo, A. Santos‐Guzman, V. Aureoles‐García, V. H. Moran, M. Sachse Aguilera, K. Markwell

**Affiliations:** ^1^ Center for Nutrition and Health Research National Institute of Public Health Cuernavaca Morelos Mexico; ^2^ Centre for Global Development University of Central Lancashire Preston Lancashire UK; ^3^ United Nations Children's Fund, UNICEF Mexico City Mexico

**Keywords:** breastfeeding commercial, breastmilk substitutes, commercial milk formula, International Code of Marketing of Breast‐Milk Substitutes, smartphone application, young child feeding

## Abstract

Almost 40 years after the adoption of the International Code of Marketing of Breast‐Milk Substitutes (‘the Code’) in Mexico, noncompliance persists. In other countries, smartphone applications for reporting Code noncompliance have proven effective. This study aimed to identify key features for the design of a public health surveillance app to monitor Code breaches and the barriers and facilitators to its use by parents and stakeholders. Semi‐structured interviews (*n* = 34) and focus groups (*n* = 14) with key stakeholders (*n* = 81), including parents and caregivers, health care personnel, representatives of academia, civil society organizations and government entities, were conducted between August and December 2023. Transcripts were analysed in MAXQDA 20 software using grounded theory ‘lite’, which emphasizes the construction of categories and concepts to explore and structure participant perspectives. Four categories were constructed from the coding process: (a) knowledge and perspectives about the Code; (b) attitudes towards reporting Code breaches and any subsequent repercussions; (c) stakeholders perspectives on monitoring the Code and (d) perspectives on the app. Mexican stakeholders supported the development of an app and associated website to monitor the Code, indicated a willingness to report breaches, and believed that a national committee and state bodies should oversee surveillance and monitoring activities of the Code. Adapting legal measures with appropriate sanctions and making infractions public were recommended. Developing an app assisted with artificial intelligence could aid the establishment of a national monitoring system for the Code, make infractions public, promote societal participation, and drive regulatory changes for commercial milk formula marketing.

## Summary

- There is a general lack of knowledge about the Code and the subsequent resolutions that only very specialized profiles recognize.
- Health professionals, CSOs, government, parents, and academics showed strong interest in the AI‐powered mobile app to report Code breaches, with participants eager to report breaches if the app is easy to use, free from conflict of interest and triggers action.

## Graphical abstract

This qualitative study explored stakeholders' views on a mobile app to monitor Breaches to the International Code of Marketing of Breast‐Milk Substitutes. Implementing a reporting app requires a campaign to raise awareness, a national monitoring system free from commercial interests, and collaboration with government, CSOs, academia, and parents.

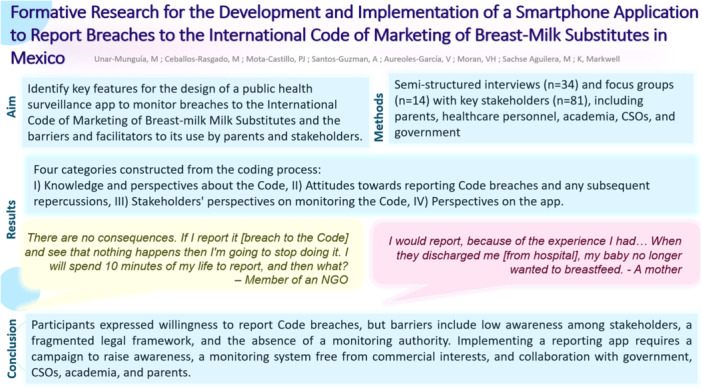

## 1 Background

The commercial milk formula (CMF) industry has negatively impacted the infant and young child feeding (IYCF) ecosystem by employing deceptive marketing strategies that exploit parental emotions and aspirations, manipulating scientific information to reshape individual, societal, and medical norms and values (Rollins et al. 2023). These practices mislead parents, creating misconceptions about the health benefits of CMF and undermine breastfeeding as the optimal choice for infants (World Health Organization WHO and the United Nations Children's Fund UNICEF 2022). The CMF industry has turned IYCF into a lucrative business, yielding annual revenues amounting to approximately $55 billion (Rollins et al. 2023). CMF is promoted to parents and health care professionals in public hospitals, traditional media, digital platforms, and educational materials (Topothai et al. 2024).

With the rise of digital platforms, social media, and artificial intelligence (AI), marketing strategies are becoming increasingly personalized, interactive (Digital Marketing Institute 2023), and widely used for the promotion of CMF, growing‐up milks and baby foods (Digital Marketing Institute 2023; Jones et al. 2022; Richter et al. 2024). CMF marketing strategies are therefore effective in normalizing CMF (Becker et al. 2022) which undermines breastfeeding globally (Richter et al. 2024; Rollins et al. 2023). For example, a study in Mexico identified numerous digital marketing strategies used by the CMF industry to influence parents' and caregivers' decisions about IYCF. These strategies include sending electronic newsletters with advertisement for CMF and baby foods via email, collecting personal information from parents, promoting online parent clubs, collaborating with or hiring influencers on social media, sponsoring webinars on infant nutrition and engaging with health professionals to promote their products (Mota‐Castillo et al. 2023). Similar strategies were also documented by the multi‐country study of the World Health Organization (WHO) on digital marketing of breast‐milk substitutes (World Health Organization 2022). A recent study in Mexico (*n* = 1074) demonstrated that parents exposed daily to digital marketing of CMF and baby food products, had a 62% lower likelihood of exclusive breastfeeding in the first 6 months of life, compared to those not exposed (Unar‐Munguía et al. 2022). Additionally, parents exposed to this marketing were more likely to feed their child CMF, commercial baby foods, and sugar‐sweetened beverages (Unar‐Munguía et al. 2022). Similarly, a study in China (*n* = 750 mothers and 300 pregnant women) showed that online engagement with CMF companies, exposure to promotions and the provision of free samples were linked to reduced breastfeeding likelihood (Zhu et al. 2023).

In 1981, following a World Health Assembly (WHA) resolution, the WHO and the United Nations Children's Fund (UNICEF) and 118 countries adopted The International Code of Marketing of Breast‐Milk Substitutes (the Code) (World Health Organization 1981). The Code is a global policy framework aimed not only at promoting breastfeeding and regulating CMF, but also at ensuring the safe use of CMF and related products such as baby bottles and teats. The Code and subsequent WHA resolutions outlines measures to protect parents from aggressive and misleading marketing practices for breast‐milk substitutes, seeking to create an environment where breastfeeding is the norm while ensuring that substitutes are used appropriately and safely when necessary. As of March 2024, only 33 countries have implemented legal measures substantially aligned to the Code (i.e., enacting laws or adopted regulations, decrees or other legally binding measures covering a significant set of provisions of the Code) (World Health Organization WHO and the United Nations Children's Fund UNICEF 2024) while 146 countries have implemented some provisions of it and 48 countries lack legal measures altogether (World Health Organization WHO and the United Nations Children's Fund UNICEF 2024). Despite these efforts, significant gaps remain in national legislation worldwide, especially in countries with moderate to weak adherence, and weak implementation of the Code has enabled the growth of CMF companies marketing strategies. With insufficient regulatory enforcement, CMF companies can continue to influence parental feeding decisions across multiple marketing strategies without accountability. Therefore, stronger national laws and global monitoring systems are urgently needed to address inappropriate CMF marketing strategies and close enforcement gaps in protecting breastfeeding practices (World Health Organization [WHO] 2023).

In Mexico, rates of exclusive breastfeeding in the first 6 months of age (34.2% in 2021–2023) (González‐Castell et al. 2024) remain far below the WHO target of 70% by 2030 (United Nations Children's Fund UNICEF and the World Health Organization [WHO] 2022). The country has implemented some legal measures that are moderately aligned with the Code, but enforcement of the legislation is weak and there is currently no monitoring system to support enforcement (World Health Organization [WHO] and the United Nations Children's Fund [UNICEF] 2024). This has enabled CMF companies to persist with promoting their products in traditional media, on the internet and social media, at the point of sale and through health care personnel (Hernández‐Cordero et al. 2019; Lozada‐Tequeanes et al. 2020; M&C Saatchi World Services 2022; Unar‐Munguía et al. 2022). Therefore, strong mechanisms for monitoring and reporting Code violations are urgently needed to ensure sanctions are routinely applied upon infringement (World Health Organization [WHO] and the United Nations Children's Fund [UNICEF] 2024).

Some countries, such as The Philippines and Myanmar, have developed reporting platforms for the Code violations. An evaluation of The Philippines' platform revealed that, whilst there was strong stakeholder interest in reporting violations of the Code, the system for implementation and enforcement needed strengthening across all levels (Reinsma et al. 2022). New technologies (Backholer et al. 2024) could be used to enhance real‐time reporting to support Governments to hold industry accountable for inappropriate CMF marketing.

In Mexico, the enforcement of legal measures aligned with the Code has been limited, and there is no quantitative data available on the number of reported violations or enforcement actions by authorities.

In response to this, the UNICEF and FHI 360 (https://www.fhi360.org/), in collaboration with the National Institute of Public Health (INSP) and other academic, civil society and key government representatives, have partnered to develop a new public health surveillance tool (a smartphone application or ‘app’ and associated website) to support the monitoring and reporting of breaches of the Code. The app is intended to have reporting capabilities of Code breaches and subsequent WHA resolutions including all products under the scope of the Code promoted in any media, physical spaces and educational materials for parents and health professionals. This study aimed to identify key features for the design of a public health surveillance app to monitor breaches of the International Code of Marketing of Breast‐Milk substitutes including all products under its scope, and the barriers and facilitators to its use by parents and stakeholders. Key stakeholders included parents, health professionals' civil society, policymakers and academia, all of whom play an essential role in breastfeeding protection. Specific objectives were to: (1) explore views, barriers and facilitators influencing parents' and stakeholders' willingness to report Code breaches through the app, and (2) identify key features the app's design, content, and functional characteristics.

## 2 Methods

### 2.1 Study Design

This qualitative study used interpretative description methodology. This approach uncovers recurring patterns or shared realities within these contexts, enabling the identification of themes and patterns among subjective perspectives related to the stakeholders' views and experiences while accounting for individual variations (Thompson Burdine et al. 2021). Data was collected between August and December 2023. To enhance the trustworthiness and validity of the study findings, we adhered to the Consolidated Criteria for Reporting Qualitative Research (COREQ) throughout the planning, execution, and reporting phases (Tong et al. 2007). For further information about the research team and reflexivity see Supplementary file 1.

### 2.2 Study Population and Sampling

Focus group discussions (FGDs) and interviews were conducted with parents (mothers and fathers) of children under 3 years of age, health care personnel (HCPs), and representatives from Civil Society Organizations (CSOs), academia, and governmental entities. These stakeholders were chosen for their distinct roles in monitoring and reporting Code violations. Parents, as primary targets of the CMF industry, are likely to encounter and report violations. HCPs, are often targeted by the industry to promote products, provide trusted guidance to families and can identify breaches within health care settings. CSOs and academia monitor adherence, advocate for accountability, and promote public awareness. Finally, government entities can implement regulatory measures to support Code enforcement and use data provided by the app for this purpose. These stakeholders are potential users of the app, hence their views on the design of the app and usefulness needed to be explored.

Potential participants meeting the inclusion criteria were recruited, as described in Table 1, following a purposeful sampling strategy (Palinkas et al. 2015) that aligned with the study's aim. Where possible, data was collected through FGDs to facilitate participant engagement and idea exchange (Braun and Clarke 2013). When organizing FGDs was impractical (e.g., participants were not close) interviews were conducted instead.

**Table 1:** Participant characteristics, recruitment strategy and total number of participants per profile.

Profile	Inclusion criteria	Recruitment strategy	Total number of FGDs completed interviews and participants
Parents (mothers and fathers) and caregivers	Mothers, fathers and caregivers of at least one child under 3 years of age living in Mexico.	An invitation (Supplementary file 2) with study details, inclusion criteria and the research team contacts was posted on INSP social media. Additionally, a Facebook search identified parenting groups, and administrators were requested to share our invitation on their group page feed. Snowball sampling. Eligible groups needed to meet the following criteria: active within 6 months, 10,000 or more members, groups that explicitly mentioned pregnancy, parenting, maternity, or feeding of children aged 0–36 months. Groups focused on selling baby products were excluded. All selected groups were required to have Mexican members. Of 32 administrators contacted, 7 posted the invitation, 1 declined and 24 did not respond.	FGDs: *N* = 6 Interviews: *N* = 1 Total participants: *N* = 21
Health care personnel (HCPs)	Medical specialists or subspecialists, nutritionists, nurses, and/or any other health care professionals in charge of care or counselling of parents and caregivers of children under 3 years of age living in Mexico.	An invitation (Supplementary file 2) to participate with information about the study, inclusion criteria and the research team contact details was displayed on the INSP social media profiles. Additionally, snowball sampling was used to enhance recruitment (16).	FGDs: *N* = 6 Interviews: *N* = 0 Total participants: *N* = 21
Government representatives	Management or senior staff of institutes and/or ministries involved in decision making on breastfeeding and/or IYCF, and public health surveillance in Mexico.	Contact details (i.e., email address) were identified through the official websites of the agencies inside the Ministry of Health (Federal) or their official social media. Invitations were sent to the director or person in charge. Representatives of these agencies at the local level were also invited. The research team held contact with some of these state level from previous projects. Yet, the invitation was extended to the authorities of other states of the country. Additionally, snowball sampling was used to enhance recruitment (16).	FGDs: *N* = 1 Interviews: *N* = 12 Total participants: *N* = 15
Civil Society Organization representatives (CSO's)	Management or senior staff of CSOs, or NGOs involved or interested in decision making on infant and young child feeding and/or the dissemination of issues related to this topic in Mexico.	A mapping was carried out to place breastfeeding groups that are currently working in Mexico, as well as organizations that have shown interest in disseminating these issues. Contact information was taken from official websites and official social media. Additionally, snowball sampling was used to enhance recruitment (16).	FGDs: *N* = 0 Interviews: *N* = 14
Academia representatives	Academics involved or interested in decision making on infant and young child feeding and/or the dissemination of issues related to this topic in Mexico.	A mapping was carried out to place academics that are currently working in Mexico, in disseminating these issues. Contact information was taken from official websites and official social medial. Additionally, snowball sampling was use to enhance recruitment (16).	FGD: *N* = 1 Interviews: *N* = 7 Total participants: *N* = 10

### 2.3 Data Collection

Topic guides (Supplementary file 2) were used during the FGDs and interviews. These guides were developed by the research team based on the supporting literature and the study's objectives. The topic guides addressed the following themes: (a) Knowledge, awareness and/or views of traditional and digital marketing on people's decisions to purchase CMF and baby foods; (b) awareness, perspectives and understanding of the Code; (c) positive and negative experiences using other mobile apps related to IYCF; (d) attitudes toward a new surveillance tool aimed at monitoring breaches related to breastmilk substitutes, specifically CMF and baby food in accordance with the Code and (e) recommendations for the usability, interface and content of the mobile app. Topic guides were amended following initial FDGs/interviews (i.e. to tailor them to the type of stakeholder and to ensure comprehensive coverage of all topics).

Participants were offered either online or face‐to‐face focus groups or interviews, and all selected the online option because they were in different parts of the country. The FGDs and interviews had an average duration of 1 h and were conducted using Microsoft Teams. Participants were advised to use headphones with a microphone, and to join the meeting in a quiet environment. At the beginning of the FGD and interview, participants were asked to complete a questionnaire via Google Forms to gather sociodemographic information (e.g., age, sex, education level, number of children, years of experience working on the field [where appropriate]). The FGDs and interviews were conducted in Spanish by an experienced research assistant (P.M.C.) from the INSP, with another team member present (A.S.G., V.A.G. and M.U.M.) to observe and ensure comprehensive coverage of the topic guide and facilitate deeper exploration of certain topics during interviews. Recording of FGDs and interviews were made using Microsoft Teams' recording and transcription feature, cross‐checked for accuracy and anonymized by the research team (P.M.C, V.A.G. and A.S.G.). Participants did not receive any compensation for their participation in this study.

### 2.4 Data Analysis

Transcripts were analysed in Spanish following the steps of the grounded theory ‘Lite’ which involves the generation of a taxonomy of categories derived from the data, revealing how concepts are related and their relative importance to the research question (Braun and Clarke 2013). This approach draws on selected elements of grounded theory, providing flexibility for identifying patterns and organizing stakeholder experiences, while maintaining systematic coding and categorization. Unlike full grounded theory, which aims to develop new theories, the ‘Lite’ version focuses on constructing categories and concepts to explore and structure participant perspectives. The ‘Lite’ version aligns with the aims of this study as it allows the creation of categories to organize the stakeholder perspectives in a structured manner. The analysis started with a codebook designed by members of the team (P.M.C., M.C.R., M.U.M. and V.A.G.), taking as a starting point the topics addressed in the interview guide, and then through a critical and reflexive reading, adding emerging issues by comparing the perspectives of each participant group. Following the analysis of each FGDs or interview, findings were discussed among the researchers. This iterative process continued until data saturation was reached, meaning no new themes emerged.

Subsequently, the codebook was refined with emerging codes identified through an agreement exercise. Two members of the research team (P.M.C., V.A.G.) independently coded data from at least one interview or FGDs from each participant group. Meetings of the research team took place were agreements and disagreements in the coding were reviewed, and discussions among the initial coders led to consensus on adjustments to the codebook definitions for a better understanding of the labels used during the coding. This iterative process continued until the P.M.C. and V.A.G. achieved at least 80% agreement, ensuring rigorous analysis.

Using the final codebook, all interviews and FDGs were coded using MAXQDA 18 software through iterative and reflective reading. Quotes that best illustrated the findings—whether representing common or unique ideas—were then extracted.

The participants' quotes presented were translated from Mexican Spanish into English by a research team member (P.M.C.) and reviewed by another member of the research team (M.C.R.). The researchers are proficient in both languages and have expertise in the subject of study. Translations were conducted by two bilingual researchers, moving back and forth between quotes, and translated data to ensure consistency. Researchers were mindful of the conceptual equivalence when translating, enabling sociocultural matching and made the adaptation of technical terms in consistency with the international literature (Younas et al. 2022). Authors (P.M.C., M.U.M., M.C.R.) evaluated the findings and agreed upon the most salient themes to be highlighted herein.

### 2.5 Ethical Considerations

This study received ethical approval from the Ethics Committee of the University of Central Lancashire (HEALTH 01013) and the Research on Ethics Committee and the Research Committee of the National Institute of Public Health in Mexico (CI:1846). Before the FGDs and interviews, participants were provided with an information sheet about the study and gave their consent before participation. In addition, the principles of respect and confidentiality of the participants were respected.

## 3 Results

### 3.1 Participant Characteristics

In total, we conducted 34 interviews and 14 FGDs with 81 participants comprising *N* = 21 parents, *N *= 21 HCPs, *N* = 15 government representatives, *N* = 14 CSOs representatives and *N* = 10 academia as detailed in Table 1. Eighteen parents completed the online demographic questionnaire. Of these 61.1% (*N* = 11) were female, all had completed a university degree (*N* = 18), 22.2% (*N* = 4) were stay‐at‐home parents, 22.2% (*N* = 4) were self‐employed and 55.5% (*N* = 10) were employed. Most parents were living with a spouse or partner (94.4%, *N* = 17) and had one child (72.2%, *N* = 13). A smaller proportion had two children (22.2%, *N* = 4) and fewer had three children (5.6%, *N* = 1). The mean age of their youngest child was 15.7 ± 11.4 months (range 0.5–32 months). Eighteen HCPs completed the demographic questionnaire, and of these 94.4% (*N* = 17) were female with a mean age of 31.8 ± 7.5 years (range 23–51 years). HCPs were dietitians (66.7%, *N* = 12), general practitioners (16.6%, *N* = 3), paediatricians (5.6%, *N* = 1), physiotherapists (5.6%, *N* = 1) and paediatric dentists (5.6%, *N* = 1), with a working experience of 7 ± 6.7 years (range 0.2–25 years).

Of the 39 representatives of academia, CSOs and government that participated in the study, 22 completed the online questionnaire. The mean age of this group was 49 ± 14 (range 25–74 years) with 72% (*N* = 16) of them being female. All the participants in this group had a bachelor's degree, and some had a PhD (31.8%, *N* = 8), MSc degree (22.7%, *N* = 5) or other postgraduate qualification (22.7%, *N* = 5) in areas related to health sciences, paediatrics, nutrition, public health, public policy and human rights. Participants of this group had a mean work experience of 13 ± 12.0 years (range 1–44 years).

Four themes emerged from the data: (a) knowledge and perspectives about the Code; (b) attitudes towards reporting Code breaches and any subsequent repercussions; (c) Stakeholders perspectives on monitoring the Code and (d) perspectives on the app. Where quotes are presented, the participant group is included alongside the data source, that is, I‐interview, FDG‐focus group discussion and … indicates edited texts. A summary of the findings by stakeholder profile is shown in Table 2.

**Table 2:** Summary of findings by stakeholder profile.

	Knowledge and perspective of the Code	Attitudes towards reporting breaches to the Code	Monitoring the Code (authorities who should be involved)	Perspectives of the app
Parents/caregivers	– It was the first time they had heard of it – Surprised that it exists and that it is breached	Willingness: Yes, if not so much time and resources needed to be invested. – If there are visible consequences	Government – As a matter for the government to resolve. Cofepris Ministry of Health	– Good reception of an application that could be perceived as useful.
				**Distribution**: Facebook, Instagram, X (formerly Twitter) and TikTok.
				– Posters in health units and services/lactation rooms
Health Care Personnel (HCP's)	– Lack of knowledge of the Code until they specialize in breastfeeding – The Code is violated daily and needs to be made law or have legal support – Ask for its update	Willingness: breaches made by CMF companies, but not breaches from colleagues	– All (parents, caregivers, industry, academia, government everyone involved must contribute to monitoring) – Government – Ministry of Health – Cofepris	– Approve the development of the application.
				**Distribution:** Facebook, Instagram, X (formerly Twitter) and TikTok.
				– Posters that incorporate a QR code in health units and services/lactation rooms
Civil Society representatives	– Lack of knowledge of subsequent resolution of the Code, so they ask for its update – Backed by national legal measures – Unification of resolutions – Lack of training regarding the Code and breastfeeding	Willingness: Yes, to anyone who fails to comply (HCPs and industry) – Mainly breaches from the industry	– Ministry of HealthCofepris, SIPINNA, CNEGSR, CENSIA, SNDIF – Interinstitutional Breastfeeding Committee	– They approve the application but warn of possible interference by the industry to sabotage it.
				**Distribution**: Social networks like Facebook, Instagram, X (formerly Twitter) and TikTok.
				– Streaming movie and series platforms now allow advertising in some plans
Academia	– The Code is only known if you work on breastfeeding or public health issues. – Lack of training for HCPs on the Code	Willingness: Yes – Training regarding the Code rather than punishment for health workers in case they break the Code	– Ministry of Health – Cofepris – Interinstitutional Breastfeeding Committee	– Celebrate the response to the need for a specific site whose responsibility is to document and process noncompliance with the Code.
				**Distribution**: Social networks like Facebook, Instagram, X (formerly Twitter) and TikTok.‐National health communication campaigns
Government representatives	– When assuming an official position or becoming involved in IYCF issues – Tool to protect breastfeeding and infant health without legal authority	Willingness: They would report through the app if there were resources to develop a processing and sanctions system to support it	– Ministry of Health – Cofepris, CENSIA, SNDIF, SIPINNA, CNEGSR – Civil Society – Interinstitutional Breastfeeding Committee	– Necessary with possible sabotage of the industry, which represents something more than just an academic exercise.
				**Distribution**: National government health communication campaigns

Abbreviations: CENSIA, Centro Nacional para la Salud de la Infancia y Adolescencia (National Center for Child and Adolescent Health); CNEGSR, Centro Nacional de Equidad de Género y Salud Reproductiva (National Center for Gender Equity and Reproductive Health); COFEPRIS, Comisión Federal para la Protección contra Riesgos Sanitarios Federal (Commission for the Protection against Health Risks); SIPINNA, Sistema Nacional de Protección de Niñas, Niños y Adolescentes (National System for the Protection of Girls, Boys and Adolescents); SNDIF, Sistema Nacional para el Desarrollo Integral de la Familia (National System for Comprehensive Family Development).

### 3.2 Knowledge and Perspective About the Code

This theme comprises three subthemes: awareness of the Code, perceptions of the Code's effectiveness, and advocacy for legal reforms.

#### 3.2.1 Awareness of the Code

Overall there was a lack of in‐depth knowledge about the Code and its resolutions among all participant groups, as one health care professional explained:

Yes, I'm… familiar with everything related to breastfeeding, promoting it, and all that. And yes, I had heard it mentioned in the hospitals where I trained, but as for having knowledge about this Code, not so much.

—A HCP, FGD3

Most parents were unaware of the Code, with only one mother having heard of it on a breastfeeding course. Some parents said they thought that it must be a recent initiative and therefore not well known among the population. A couple of mothers mentioned that there should be more promotion of the Code, as they had observed breaches, particularly in social media and private clinics:

He [paediatrician]always had the same, well, the formula milk information back there, in fact, that was why we decided to change paediatrician and well, they violated our right to skin‐to‐skin contact… Since on his [son] first day of life he was given a bottle without my consent. So yes, we have been bombarded with this advertising on social networks, on Facebook, and Instagram.

—A mother, FGD2

Breaches had occurred without parents being aware that they were unlawful at the time. Some parents expressed frustration, as one reported not being offered the opportunity to breastfeed while in the hospital, while others mentioned that paediatricians or other health professionals had promoted formula milk to them:

The paediatrician told me to give formula to my daughter, he gave me free samples. I did not know this was prohibited, but he did give me 2 samples of Enfamil.

—A mother, FGD1

#### 3.2.2 Perceptions of the Code's Effectiveness

While participants from academia, government and HCPs, were aware about the existence of the Code, some of them claimed that they were unaware of it until they specialized or worked on breastfeeding issues:

I don't remember seeing it in the bachelor's degree in a formal way… until I began to specialise in paediatrics…. but really, how small a part of the population knows about it… not many of my nutritionist colleagues who are not dedicated to this part [IYCF]…I am sure that they carry out practices that they should not have, or that could violate it [the Code].

—A HCP–FGD2

The Code was perceived by some HCPs and representatives of government and CSOs as an important tool for protecting breastfeeding and infant health but without legal or punitive authority:

Many other types of reforms and legal adjustments still need to be made so that the Code really works as expected.

—A HCP–FGD2

The relevance it [the Code] has for both decision‐makers and companies are fundamental. I think the challenge is that it is a compendium of good intentions. In other words, there is currently no legal instrument that obliges the industry or the government itself in our country to comply with the criteria established in the Code.

—A government representative, I 4

#### 3.2.3 Advocacy for Legal Reforms

There were repeated calls by CSOs representatives and academics to either create specific regulation to protect breastfeeding or to include it as part of the regulations established by the Mexican Ministry of Health in the Official Mexican Standard NOM‐043‐SSA2‐2012 regarding health services and the promotion and education for health in food matters (Secretaría de Salud 2013), and Official Mexican Standard NOM‐007‐SSA2‐2016 for the care of women during pregnancy, childbirth and the postpartum period, and of the newborn (Secretaría de Salud 2016). A representative of a CSO emphasized the priority of establishing the Code as a law and reforming the Regulation for Health Control of Products and Services (Gobierno de México 2022a) and the General Health Law on advertising (Gobierno de México 2022b):

I believe that the most urgent are the regulation of health control of products and services and the regulation of the General Health Law on advertising. It would be necessary to analyse the General Health Law, of course, to be able to provide a basis for something, but operationally I believe that these two instruments would be the ones that require urgent reform.

—A CSO representative, I 1

Academics also advised that reforms to other laws, such as the labour law to extend maternity paid leave, could complement these national legal measures as it would also have an impact on the mother's ability to breastfeed. Industry was identified by some representatives of CSOs and government as an opponent of these legislative changes:

A few months ago, at a national paediatric congress, one of the sponsors was [CMF brand] … while there are a series of constant systemic violations, these violations allow them to try to interfere with these policies… what happens if a [Government Organization] is given a solution [by industry] to its problems… and [Government Organization] accept it? … My scope is limited to issuing memos, giving instructions to state DIFs to prevent conflicts of interest, discussing the conflict of interest as a problem. However, at some point, there is no authority to enforce a sanction because there is no regulation being violated. So, the first step is that we need a regulation so that we, as officials, can seek to apply a sanction or use our authority to prevent these types of situations.

—A government representative I 3

### 3.3 Attitudes Towards Reporting Breaches to the Code and any Subsequent Repercussions

Five subthemes underpin this theme: Willingness to Report Breaches to the Code, Facilitators to Reporting, Barriers to Reporting, Who Should Be Reported, and Sanctions.

#### 3.3.1 Willingness to Report Breaches to the Code

All participants indicated their own willingness to report breaches to the Code. However, some stakeholders suggested that only strong breastfeeding advocates would report breaches. In this context, a representative of the government referred to them as ‘extremists’:

Hmmm. I don't think so [people would not report breaches to the Code], you see, probably a few extremists would do it [report], but not most of the workers will do it, but there are always extremists, right? So, if they adhere closely to an instruction, they probably would, but I don't think there will be a lot of reports

—A government representative, I 12

In contrast both CSO representatives and parents expressed their willingness to report any breaches of the Code that they may encounter, contingent upon knowing where to report and being assured that their report would prompt action:

Yes, I would do that too, report it. Obviously, once that I am informed and that I know that Code… The important part for me would be that notifying [a breach] was very easy and very brief, but yes, obviously yes… I would notify… I feel that when the same thing happens on the street, with certain crimes… if you report it [problem]and see that they immediately come and fix it and it won't happen again…

—A father, FGD1

#### 3.3.2 Facilitators to Reporting

An academic expressed that their reason for reporting would be to ethical responsibility. Similarly various participants indicated their willingness to report as a means of protecting public health. An impactful outcome as a result of a Code violation report was identified as a facilitator to reporting breaches to the Code. Representatives of CSOs noted that parents might be motivated to report breaches if they felt part of a community. Moreover, perceptions about the accessibility of the reporting process and the time and effort required to report a breach could influence participants' willingness to report noncompliance with the Code, especially if there were clear instructions of what can be reported. Some mothers reported experiences that may constitute violations of the Code. For example, they were not given their newborns to breastfeed in the hospital, which they felt could be a result of pressure to use formula instead. Such experiences would encourage them to report potential breaches of the Code. Additionally, some participants perceived that women's empowerment would also encourage reporting. One mother said that she would report a breach to protect women from being discouraged from breastfeeding:

I would report it as well…many mothers have the dream of breastfeeding, but because of so much advertising and so many myths, well, then, I mean, if even the paediatrician recommends it [CMF], you think, ‘wow, then I guess it's the best,’ right? … so, I think it would be great to start implementing this law, and it would be really cool to be able to report it to avoid disrupting breastfeeding.

—A mother, FGD2

#### 3.3.3 Barriers to Reporting

Potential barriers to reporting were also identified. Academics and CSO representatives noted that public lack of awareness about what constitutes a breach could hinder reporting. Additionally, some representatives of academia and CSOs, believed that conflicts of interest within medical or nutritional communities may deter people from reporting. In relation to this, some HCPs and CSO representatives reported that either them or their colleagues were currently accepting funding or support/sponsorship from the CMF industry to assist with meetings and events organized by CMF industry and to share updates of their products and promote their use:

Well, for many years here in Mexico City, there was an event at the National Auditorium called ‘Selected Topics in Nutrition,’ … It was completely, completely sponsored by Nestlé, and at the end of the day, as a participant, you never really realized the impact it had because they would give you a backpack and tons of stuff.

—A HCP, FGD4

**Figure 1:**
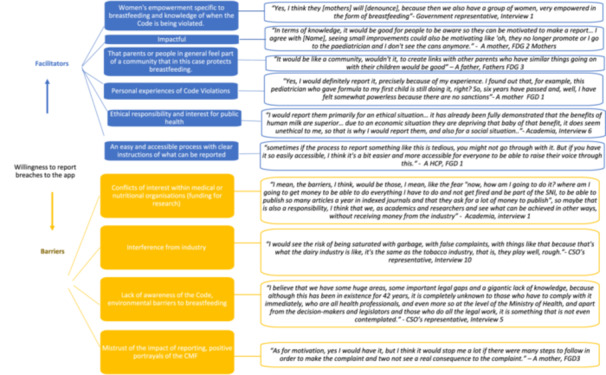
Identified potential barriers and facilitators to report breaches to the International Code of Marketing Breast‐Milk Substitutes.

A representative of a CSO expressed their concern that there was the potential for industry interference, such as the platform being overwhelmed with false complaints. Moreover, all participant groups felt that if reporting was felt to be ineffective or they would not trust the agency where report was made, it would discourage them from reporting. The barriers and facilitators to report breaches to the Code are presented in Figure 1.

#### 3.3.4 Who Should Be Reported

Participants expressed different opinions on which individuals and/or entities should be reported through the app in case of Code violations. Some argued that app users should be able to submit complaints against all parties involved—such as manufacturers, HCPs or health facilities, and points of sale, since the Code outlines specific guidelines for each entity. This represented a dilemma for some of the participants, particularly the HCPs, who felt uncomfortable reporting colleagues or their employers if they do not comply with the Code. Other participants condemned the actions of the industry but not those of HCPs. They perceived that if HCPs engage in conduct contrary to the Code, it is because of lack of training or due to pressure and incentives (e.g., funding, training and conferences) exerted by the CMF industry, who should ultimately be the ones being denounced:

I understand that to a certain extent the doctor can also be a victim of the same thing, but those who are not victims are the industry. They do know what they are doing and if they know why they are doing it, then I think it would be better to report the brand that is doing these practices, rather than the doctor or the hospital.

—A father, FGD3

#### 3.3.5 Sanctions

The nature of what penalties or sanctions should be applied for noncompliance with the Code directives was also debated, particularly concerning sanctions for HCPs. One viewpoint recognized that the Code includes specific calls for action for HCPs such as maintaining clear professional boundaries by refusing gifts, favours or hospitality that could compromise their impartiality or influence their professional judgement. Another HCP clarified that the objective is to stop commercialization, making the approaches and practices the responsibility of the industry, which should be the only entity sanctioned. Therefore, HCPs felt that the solution to prevent violations to the Code was improved training, not sanctions. A HCP emphasized that the goal is to raise awareness and protect breastfeeding rather than to harm individuals or institutions:

it's perhaps more about raising awareness that these types of actions, whether by people who can influence mothers or other health professionals, or institutions that can also be influential, should be considered since sometimes these things can harm breastfeeding. Maybe not focusing so much on harming the person or, in this case, the institution, but rather on making them aware that it has an impact on breastfeeding.

—A HCP, FGD1

CSOs and government representatives highlighted that the support of HCPs would be lost if sanctions were given to this group. Another suggestion was to create a typology of noncompliance to better determine fines, especially for industry:

Well, consequently, I think, for example, I currently understand that there is a fine… of approximately $100,000.00 MXN [5,600 USD] for non‐compliance with the Code. It is not the same consequence to give a fine to Nestlé than to a paediatrician on his own practice… I believe that there should be proportional sanctions depending on who is doing it [infringement]… maybe a monetary fine and having certain, I don't know, maybe 3 consecutive fines is already a sanction for suspension of, well, some closure of the practice…

—A government representative, I 5

Some representatives of academia and CSOs stressed the need to publicly expose those do not comply with the Code. One of them stressed that the financial penalties should be clearly announced and extensively covered by all media outlets and social media, to show that violations are being taken seriously and that the government is acting:

An outstanding financial penalty, publicly documented, that gets covered by all the media. Now it's time to use social media for this, so people can see that penalties are being enforced, that they are indeed violating the code, and that the government is taking action

—CSOs representative, I 12

### 3.4 Stakeholders' Perspectives on Monitoring the Code

Two subthemes form this theme: Suggested Organizations for Monitoring and Funding Concerns.

#### 3.4.1 Suggested Organizations for Monitoring

When asked about the way in which a new monitoring system should be set up, organizations such as PROFECO (Federal Consumer Attorney), COFEPRIS (Federal Commission for Protection Against Health Risk), the Ministry of Health, as well as other national institutions such as the SNDIF (National System for the Integral Development of the Family), CENSIA (National Centre for Child and Adolescent Health) and SIPINNA (National System for the Protection of Children and Adolescents) were suggested as the bodies that should take on the responsibility of monitoring the Code compliance. A representative of the government mentioned that monitoring should be a societal responsibility:

Monitoring, this should be done by everyone, not just the health sector like us, the Ministry of Health. Civil society should also be involved. Currently, the civil side, as I understand it, is supported a lot by La Leche League in these community reports of code violations concerning substitutes…. So, who should be responsible? Everyone, that is, society as a whole, but we, as part of the government, should also establish channels for reporting violations.

—A government representative, I 1

Whether a new department or committee should be established to deal with Code monitoring and enforcement polarized responses. Decision makers stated that it was not the time for new institutions to emerge, but rather collaboration between existing ones should be improved. On the other hand, academics thought the creation of an entity dedicated solely to the Code would be useful, particularly when integrating new technologies such as AI to help identify noncompliance:

I think we can use these same [artificial] intelligence and machine learning tools to be able to do more realistic regulation or monitoring. So, I think we should use the same instruments that the industry uses to commercialise, we should use them to monitor, but the one that sanctions should be the State…

—An Academic, FGD1

For enforcement activities relating to noncompliance, academics and CSO representatives proposed that trusted and autonomous international organizations, such as UNICEF and the WHO, could lend credibility to the system. These suggestions were based on concerns regarding the potential conflicts of interest within governmental agencies, which may have affiliations with the CMF industry. Respondents emphasized the need for unbiased oversight to ensure the effective implementation of the Code:

And it should be, as I said, an external organization, someone who really knows the issue, someone genuinely interested in ensuring compliance. Obviously, with the backing of the WHO, UNICEF, and all those international organizations because it gives you much more weight, but an external organization would be excellent in influencing all these sectors.

—A CSO representative, I 3

#### 3.4.2 Funding Concerns

Some of the participants expressed a shared concern about the need of sustained funding to support the monitoring of the Code and added how conflicts of interest could compromise the objective of this initiative:

The issue of resources is always a challenge; there's never money for these topics… sustainability could be one of the challenges. Another issue is that toes are stepped on when it comes to conflicts of interest… the industry itself might want to place obstacles with the health department, possibly using blackmail like ‘oh, then we're not going to support this or that anymore’ …. They[industry] might come forward and say, “Sure, we'd be happy to sponsor the funding for this application or part of it so that we can be monitored and prove that we're doing everything according to the law…

—A CSO representative, I 1

### 3.5 Perspectives on the App

This theme is organized into three subthemes: App Features and Content Preferences, Promotion and Awareness, and Empowering Citizens Through Reporting.

#### 3.5.1 App Features and Content Preferences

**Figure 2:**
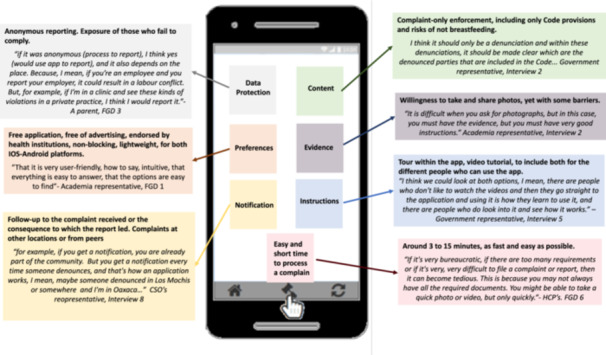
Participants' views on the features and characteristics that the app should have when reporting the breaches to the code.

Participants gave their views on the features and characteristics that the app should have to encourage them to download and use it. A summary of these features and characteristics are presented in Figure 2.

Participants mentioned facilitators and barriers for the app's use. They noted that widespread phone and internet access facilitates use, but a barrier to those with limited or no access to the internet. They emphasized the importance of a well‐designed, low‐storage app that doesn't slow down their phones. Additionally trusted institutional logos and the absence of any conflict of interest would increase their confidence in reporting Code breaches using the app:

That is easy to use, that doesn't take up too much space on the phone… And really, if it's an application that is endorsed by the health sector, in this case the National Institute of Public Health, then there would be no doubt that it is something, obviously, with a solid foundation.

—A HCP, FGD1

Overall, participants welcomed the existence of an app as a dedicated tool that citizens could use to report breaches of the Code:

It's going to be excellent, because several people have already asked me where they are going to go or how they are going to report these types of irregular situations, and if it's in a digital way it's excellent, because everyone will have it on their mobile phone… will simplify things a lot, and then people will be more cautious, it's definitely an excellent initiative, we already needed something like that.

—A government representative, I 14

Views about the app content varied among participants. Parents primarily expressed that they would value an app that provided them with useful information in addition to reporting breaches to the Code. One mother suggested that a system that tailored information to the stages of their child's development would be helpful. Another mother emphasized on the importance of a constantly updated information to keep users engaged. Many parents also expressed the idea of an app that would allow them to foster some sense of community:

I believe that it should not only serve to report, but also to create a bit of community among moms who are in the same process and supporting each other. I mean, it should offer the opportunity to interact with other moms and include other general content. Like articles, maybe to promote breastfeeding or address common concerns among moms, or other content that would be of interest to those who might want to report.

—A mother, FGD3

Academia, government, CSOs representatives and HCPs also had varied views as to the content of the app. Some of them believed that the app should remain focused on Code reporting, the importance of breastfeeding and the risks of CMF:

To avoid overwhelming one platform with a single issue, perhaps we should continue focusing on ongoing training for both staff and the population overall. If there is a platform solely for filing complaints and reports, it would help keep these two topics separate, even though they revolve around the same issue.

—A Government representative, FGD1

However other participants believed that it should also include content attractive to parents such as learning materials about the child's development, breastfeeding and complementary feeding and other parent's lived experiences. Additionally, a health professional thought that it would be useful for the app to include trustworthy information about breastfeeding and information about professional courses or research articles:

I think that if it were an app just for reporting, you wouldn't lose sight of the objective, right? People would know that when they see something, they can use it for that, because that's the purpose of the app. But on the other hand, especially since I work with moms who breastfeed, pregnant moms, they're always looking for information in many ways about breastfeeding, myths, and all this. having an app that includes evidence‐based information, I mean, good information about breastfeeding could be a way to reach mothers and get them to use it more,

—An academic, I 1.

Some representatives of academia and CSOs believed that the ‘dairy/milk industry’ [as some participants called the CMF industry] would be one of the main opponents of the app's launch. They listed different strategies that could be used by those wishing to disrupt the app's use, such as hacking, saturation of the service by sending multiple erroneous complaints, legal appeals and disinformation campaigns:

I am an alarmist, but one of the dangers is that they will be saturated with bots. The dairy industry is very cynical… I would see the risk that they would saturate it with rubbish, false complaints, things like that because the dairy industry is like that, it is the same as the tobacco industry. They play well, rough.

—CSO representative, I 10

#### 3.5.2 Promotion and Awareness

Participants suggested that the app, together with awareness raising about the Code, should be promoted in social media, particularly Facebook, Instagram, X (formerly Twitter) and TikTok, streaming platforms, traditional media (TV, radio, newspaper), paediatric health centres and breastfeeding rooms. It was also suggested that posters, containing a QR code to where the application can be downloaded, could be placed in health units that serve children under the age of three and their families, as well as in breastfeeding rooms in places such as cafes and airports:

There are people who are watching Netflix all the time, so you have to have a commercial there [laughs], there are people who are much more on the networks… like Instagram TikTok, so that's where you must have a commercial…

—A CSOs representative, I 2

#### 3.5.3 Empowering Citizens Through Reporting

A representative of a CSO, stated citizen reporting through the proposed app could redress power imbalances, enabling citizens to make industry more accountable for their actions:

The power of having an app in your literal hand is a mechanism for balancing forces and in terms of breastfeeding it is a tool for breaking down barriers and making more equal participation of the citizenry in terms of feeding and nurturing. The difference, I repeat, would be that it is protecting the population through a new culture of complaint.

—A CSO representative, I 8

## 4 Discussion

This study generated new knowledge to inform the design and usability of a new mobile app to monitor breaches to the International Code of Marketing of Breast‐Milk Substitutes and explored the perceived barriers and facilitators influencing the app's future adoption by parents and key stakeholders.

Our findings showed a general lack of awareness about the Code among key stakeholders, particularly parents and HCPs. This trend consistent with studies in Mexico (Hernández‐Cordero et al. 2023; Mota‐Castillo et al. 2023; Mota‐Castillo and Urizar Pastor 2023) and other countries (Čatipović et al. 2022; Doherty et al. 2022). Even among participants familiar with the Code, many were unaware of subsequent WHA resolutions addressing emerging concerns and extending the original provisions (54 World Health Assembly [WHA] 2001; 69 World Health Assembly [WHA] 2016; 78 World Health Assembly [WHA] 2022; Theurich 2018). This lack of awareness emphasises the need for ongoing education to ensure comprehensive understanding and adherence to the Code.

The interviews revealed that participants had witnessed a broad range of noncompliance with the Code, including CMF marketing and promotional activities targeting mothers, families, HCPs and the general public, which has been addressed in the 2016 WHA resolution (69 World Health Assembly [WHA] 2016). Similar violations have been documented in studies from Mexico, in both digital and traditional media channels. (Hernández‐Cordero et al. 2019; Lozada‐Tequeanes et al. 2020; Unar‐Munguía et al. 2022). For instance one study found that 84.4% of women were exposed to breast‐milk substitutes promotion, 10.9% received a sample and 18.8% of health providers reported contact by CMF companies to promote their products (Lutter et al. 2022). Additionally, 98% of retail outlets promoted breast‐milk substitutes, and all products made nutrition or health claims (Lutter et al. 2022). Another study found that 93.9% of a sample of parents with internet access were exposed to CMF digital marketing (Unar‐Munguía et al. 2022). Our study also found that many HCPs and their colleagues accept sponsorship or support from the CMF industry, a conflict of interest presents a critical challenge to the effective implementation of the reporting system as documented previously in other study in Mexico (Mota‐Castillo and Urizar Pastor 2023) The CMF industry often portrays formula as a premium product in their marketing (Rollins et al. 2023) which may hinder parents' understanding of the potential negative implications of CMF. While parents in our study recognized the inappropriateness of such practices, many were unaware that these actions constitute breaches of the Code. Increasing parental awareness about the Code and empowering them to identify violations such as online marketing or distribution of free samples, could position them as key agents in reporting breaches.

To effectively support the monitoring and enforcement of the Code, stakeholders emphasized the need to strengthen Mexican legislation. Academia, CSOs and government representatives agreed that current laws, such as the General Health law, require amendments to include the Code's provisions and regulate CMF marketing without conflicts of interest. The International Baby Food Action Network (IBFAN) has also highlighted the need for legal changes in Mexico to prohibit the promotion, advertising and sponsorship of CMF and related products. Further, educational materials should highlight the superiority of breastfeeding and the risks of CMF. Health systems and personnel should be required to support breastfeeding and prohibit CMF manufacturers' influence. Product labels must not discourage breastfeeding or depict infants, mothers, or toys; in ways that promote formula use. Additionally, monitoring and evaluation processes should be implemented to prevent conflict of interest to ensure the Code compliance (Alianza por la Salud Alimentaria 2017).

Academics and CSOs also proposed legislative changes amendments to existing laws, for example to the National Regulation for Health Control of Products and Services (Gobierno de México 2022a), the General Health Law on advertising (Gobierno de México 2022b), the labour law to extend paid maternity leave (Gobierno de México 2024b), and the Mexican official norms regarding the content and labelling of CMF and food for infants and young children (NOM‐131‐SSA1‐2012) (Secretaría de Salud 2012), the norm for health services and the promotion and education for health in food matters (NOM‐043‐SSA2‐2012) (Secretaría de Salud 2013), the norm for the care of women during pregnancy, childbirth and the postpartum period and of the newborn (NOM‐007‐SSA2‐2016) (Secretaría de Salud 2016). Participants also suggested revisiting the proposed norm for the promotion and protection of breastfeeding (PROY‐NOM 050‐SSA2‐2018) (Secretaría et al. 2018a). This draft norm did not progress, and in the file submitted to the National Commission for Regulatory Improvement (CONAMER), industry representatives from the chambers of milk and pharmaceutical industry provided feedback. In their official correspondence, these representatives raised concerns that the provision could harm economic competition by discriminating against manufacturers of breastmilk substitutes and potentially contravene Mexico's existing regulatory framework (Secretaría de Salud 2018b).

Some progress towards stronger legislation for promoting and protecting breastfeeding in Mexico and political commitment to regulate CMF marketing has been made in recent years. During the World Breastfeeding Week in 2023, the National Breastfeeding Law Initiative was presented in Mexico (Bonvecchio and Unar 2023; Cámara de Diputados LXV Legislatura 2023) to support breastfeeding promotion and enforce the Code, although it was not subsequently approved. In November of the same year, the General Law on Adequate and Sustainable Food was approved and published in April 2024 (Gobierno de México 2024a). This law has a specific remit for promoting breastfeeding and adequate complementary feeding, training health care personnel, avoid discrimination against breastfeeding women, adequately implementing the Code and monitoring policies. This established a significant precedent of interest in public nutrition in Mexico (Canal del Congreso 2023). However, the regulation derived from this new law must derogate any other legal measures that contravene the Code or modify existing ones to fully align with the Code, which can be a barrier to the proper implementation of the Code due to the difficulty of aligning many legal instruments that have provisions of the Code in a fragmented manner. In addition, at the 77th WHA in May 2024, Brasil presented a joint statement co‐sponsored by Mexico with other countries to support the WHO Guidance on regulatory measures to restrict digital marketing of breastmilk substitutes and intends to prepare a draft resolution to be tabled at the 78th WHA approved by the Executive Board on February 2025 (WHO 2025). If adopted, this resolution will support legislation changes, and the adoption of regulation measures and thus contribute to compliance with the Code (World Health Organization [WHO] and the United Nations Children's Fund [UNICEF] 2022).

The establishment of a committee or body dedicated to monitoring the Code proposed by academia and CSO representatives in our study, could ensure transparency, monitor compliance and coordinate intersectoral efforts. Our findings support previous work that called for the implementation of a ‘National Breastfeeding Committee’, constituted by representatives from multiple sectors. The committee would coordinate the development of public policy and promote oversight and transparency of how policies are implemented (Cobo‐Armijo et al. 2017) and be responsible for the monitoring of advertising of CMF. Currently, monitoring of the Code falls under the responsibility of COFEPRIS to monitor the application of the Code. However, there are currently no specifications on how the monitoring system should operate (Gobierno de México and Centro Nacional de Equidad de Género y Salud Reproductiva 2022).

Some participants from academia and CSOs recommended using AI as another tool for monitoring CMF marketing. Such innovative tools could support monitoring of the Code specially in the Latin American context, where, as reported by stakeholders in our study, there are difficulties in monitoring adherence to the Code mainly due to lack of funds, trained personnel and technical support (Organización Panamericana de la Salud 2016). AI has the potential to automate large‐scale monitoring of unhealthy food marketing, addressing resource constraints and policy enforcement challenges. AI‐driven approaches can enhance surveillance by improving real‐time detection of digital marketing strategies and policy violations. Given the industry's increasing use of advance AI techniques to market unhealthy foods and circumvent regulations, AI‐based monitoring systems offer a competitive solution to ensure effective policy enforcement (Olstad and Lee 2020).

The Virtual Violations Detector (VIVID), a virtual platform used in Vietnam to monitor breaches to the Code (Backholer et al. 2024; Innovation Incubator at FHI 360 2023) is an example of such AI technologies can streamline surveillance, particularly in digital media, while reducing the resource burden. VIVID autodetects public pages of websites, social media channels, and shopping platforms for Code violations, and has built‐in functions to integrate monitoring findings with an enforcement action tracker for countries with national measures. The government of Vietnam has been using VIVID to assist in monitoring and tracking enforcement of national measures since 2022. VIVID also encourages citizen's participation by providing both AI‐powered and crowd‐based reporting options for members of the public to report breaches in both digital and non‐digital settings in real time. VIVID has real‐time data analysis functions to provide AI‐generated reports and insights to optimize use of monitoring findings for advocacy, awareness‐raising, and research (Backholer et al. 2024). During its beta phase in Australia, Hong Kong, Malaysia, Nigeria, the Philippines, Singapore, the UK, the USA, and Vietnam, VIVID identified nearly 30,000 suspected Code violations on digital platforms (Innovation Incubator at FHI 360 2023). The organizations currently working towards the potential app for reporting breaches to the Code investigated in this study, are planning to integrate similar technology to VIVID into the app, along with the associated website. that will feature AI monitoring of digital media, empower stakeholders to report breaches in real time, thereby reducing the burden of human and financial resources for this task. As requested by participants in our study, the proposed app could foster the sense of community through providing educational resources free from conflict of interest for parents, caregivers and health professionals, facilitating communication and incentivizing users' engagement to report Code's breaches by making transparent the complaints received, their follow‐up and any sanctions issued.

Participants of our study also emphasized that visible actions and sanctions for noncompliance would motivate them to report breaches, as they would feel their efforts were not wasted and some of them gave suggestions as to the nature of the sanctions that should take place as a consequence of breaching the Code. Some participants recommended that HCPs and companies should be issued with fines for noncompliance. Similar fines to industry have been implemented in Brazil, where the amount of the fine is determined by the type of violation (Müller et al. 2021). Members of academia and CSOs suggested that companies that do not comply with the Code should be ‘named and shamed’ with consequent damage to their reputation. This aligns with the Pan American Health Organization who have recommended that companies that fail to comply with the Code must be exposed, so they lose credibility and trust (Organización Panamericana de la Salud 2011). This approach is similar to one already taking place in Mexico, where every quarter, COFEPRIS publishes the lists of people, institutions or brands that fail to comply with the General Health law. This law regulates the right to health protection that every person has under the terms of the Political Constitution. It establishes the foundations and modalities for access to health services, distributes competencies, and determines cases of concurrent jurisdiction between the Federation and the federative entities in matters of general health. Although violators to the Code have not been shown (Comisión Federal para la Protección contra riesgos sanitarios Gobierno de México 2023).

In our study, the CMF industry itself was identified as a potential barrier to the introduction and maintenance of a monitoring and reporting system and the legislative changes that would be needed to support it. As mentioned by Rollins; creating an enabling policy environment for breastfeeding that is free from commercial influence will require greater political commitment, financial investment, CMF industry transparency and sustained advocacy for this new system or monitoring (Rollins et al. 2023).

A monitoring system, like the one proposed in Mexico, has been implemented in The Philippines (Reinsma et al. 2022) and consists of three strategies: a website, an SMS messaging system, and a mobile application. Notably, their mobile application includes an offline functionality to address issues related to limited internet connectivity, a challenge noted by some of the participants in our study. Therefore, incorporating offline functionality into the application's design is crucial for addressing similar challenges. One of the key lessons learned in the work of the Philippines was to consider the investment capacity of countries to manage the resources required to provide feedback to citizen reporting. The concern about financing was also expressed by CSOs and academia representatives in our study. Strengthening public funding mechanisms and seeking independent, conflict‐free sources of financial support are essential to ensuring the long‐term success and integrity of initiatives aiming to protect breastfeeding. Further exploration is needed to understand how sustainable funding models can be developed, mitigate conflicts of interest and ensure that financial support aligns with promoting compliance with the Code.

### 4.1 Implications

Stakeholders show willingness to report breaches to the Code if provided with an easily accessible mobile tool supported by trusted organizations or authorities who follow up on their reports, and sanction and expose violations, mainly from CMF industry. Given the widespread lack of awareness about the Code, ongoing breaches and persistent conflict of interests between HCP and CMF industry, this study emphasizes the need to train key stakeholders (i.e., HCPs, CSOs, parents, decision‐makers) and to increase public awareness about the Code. This should be carried out before or alongside the introduction of an app to monitor and report breaches to the Code (World Health Organization 1981). Future research could explore educational strategies to increase awareness about the Code among parents and key stakeholders. In this regard, the National Institute of Public Health in collaboration with the Mexican Institute of Social Security, the largest health provider in the country, and UNICEF have developed a free virtual course on the Code so that HCPs, parents and anyone interested can learn about it (Instituto Mexicano del Seguro Social 2024). This course should be widely promoted to expand the understanding of the Code among stakeholders.

Changes in Mexican legislation are needed to fully align to the Code and subsequent resolutions, and, it is important to clearly state the faculties of the institution that will be in charge of monitoring compliance with the Code and national legislation (i.e. COFEPRIS), and implementation of sanctions for infractions, and the establishment of a committee free from conflict of interest that will help in the surveillance process and follow up on the reports, which could be the interinstitutional breastfeeding committee and the implementation of a breastfeeding strategy such as the one promoted in 2014–2018 (Secretaría de Salud 2014).

It has been documented that, in the Latin American context, there are difficulties in monitoring adherence to the Code mainly due to lack of funds and technical support to the national organ in charge of this task, a situation that could arise in Mexico (Organización Panamericana de la Salud 2016). Surveillance using new technologies could assist in monitoring digital media, which is quickly becoming the main channel for promoting CMF, thereby reducing the burden of human and financial resources for this task.

### 4.2 Strengths and Limitations

The findings of this study relate specifically to a Mexican population. However similar Code breaches have been reported in other Latin American countries (Bustos and Vasquez 2022; Lutter et al. 2022; Piaggio et al. 2023) alongside regional policy challenges in enforcing and monitoring adherence to the Code. Similar to other countries in the region, such as Argentina (United Nations Children's Fund UNICEF Argentina 2023), there has been recent government interest in implementing laws that protect breastfeeding which may resonate with other studies in Latin America. For example, Mexico's sugar tax influenced similar policies across Latin America, highlighting how regional measures can inspire broader action (Carriedo et al. 2021).

Our study provided interesting insights into the potential motivators of key stakeholders to use an app to report breaches to the Code, recruiting a broad range of stakeholders to provide a comprehensive viewpoint. However, participant data was collected based on a hypothetical scenario involving an app that is currently been developed. This limits the findings as it is uncertain if positive attitudes toward reporting will translate into actual reporting once the app is released. This limitation may impact the reliability of our findings, as hypothetical scenarios may not fully capture the complexities of real‐world behaviours. Once the app has been developed, a pilot study could be conducted to assess its practical effectiveness. In addition, many of the stakeholders we recruited held job roles that may predispose them toward valuing the importance of protecting breastfeeding and promoting adequate IYCF practices potentially leading to over ‐exaggeration of the positive reception towards the app, which may not be reflected in the general population. Further, parent participants were more highly educated compared to the average education level of the country's population and therefore our findings may not reflect the experiences of the wider population or those with different views on breastfeeding. Additionally, as all participants opted for online interviews or FGDs, potential biases related to participant engagement or the depth of the responses in a virtual setting cannot be ruled out. However, despite these limitations, most parents were unaware of the Code, highlighting the need for awareness raising in the general public together with HCP training, to best support the implementation of an effective monitoring and reporting system to minimize the harmful inappropriate marketing of CMF. Lastly, the translation of the quotes from Spanish to English could have introduced interpretation bias as no independent review or back‐translation was implemented.

## 5 Conclusion

Overall, the participants in our study reported a willingness to report breaches to the Code. However, important barriers to the app's implementation includes a lack of awareness among stakeholders about the Code, a fragmented legal framework that does not include all provisions of the Code, and the absence of an authority with the faculty to monitor and sanction violations. Therefore, the implementation of an app to report breaches should be carried out together with a campaign to raise awareness about the Code and the establishment of a national monitoring and enforcement system which involves input from the government, CSOs, academia, parents and other key stakeholders that are free from commercial interest. It is important to continue to conduct research on improvements to monitoring of the Code. Future research should focus on optimizing systems to monitor the Code, and, if a tool is developed, studies using an implementation science approach could provide lessons and serve as a model for similar systems in Latin America.

## Author Contributions

M.U.M., M.S.A, M.C.R., V.H.M. and K.M. conceptualized the study and contributed to the study design. M.C.R., M.U.M., V.H.M. and K.M. contributed to the grant application. M.U.M. and M.C.R. led the study. M.U.M, M.C.R., A.S.G., P.J.M.C. and V.H.M. were involved in the instrument design and informed the study protocol. Data collection was mainly managed by P.J.M.C., a team member (A.S.G., V.A.G. and M.U.M.) was present to observe the interviews, ensuring comprehensive coverage of the topic guide and facilitating deeper exploration of certain topics. V.A.G. and A.S.G. reviewed transcripts for accuracy. P.J.M.C., V.A.G. and M.C.R. coded the data. P.J.M.C., M.U.M. and M.C.R. contributed to the conceptual design of the analysis and the interpretation of the results. P.J.M.C. and M.C.R. developed the first draft of the manuscript. M.U.M. and V.H.M. completed the manuscript. M.S.A. and V.H.M. provided important intellectual content. All authors reviewed, revised, provided feedback, and approved the final manuscript.

## Conflicts of Interest

The authors declare no conflicts of interest. One of the authors (VHM) is a Senior Editor of Maternal & Child Nutrition. They were not involved in the peer review or editorial decision‐making process for this article.

## Supporting information

Supporting information.

Supporting information.

## Data Availability

The data that support the findings of this study are available from the corresponding author upon reasonable request.
