# Supplementary material for: Formative Research for the Development and Implementation of a Smartphone Application to Report Breaches to the International Code of Marketing of Breast‐Milk Substitutes in Mexico
Source: Matern Child Nutr. 2025 Mar 18;21(3):e70014. doi: 10.1111/mcn.70014 (PMC12150152; doi:10.1111/mcn.70014)
Supplement: Supplementary file 1 — Supporting information. [file MCN-21-e70014-s001.docx]

# Researcher Characteristics and Reflexivity:

What characteristics might have influenced the researchers' perspectives?

Dr. Mishel Unar-Munguia (M.U.M.), PhD in Population Nutrition, MSc in Health Economics, BSc in Economics. Mainly quantitative researcher, with 19 years of experience in research in the area of Public Health Nutrition and Health Economics. Researcher at the National Institute of Public Health. Research interests: maternal and child nutrition, design and evaluation of interventions and programs, sustainable diet and nutrition economics.

Dr. Marena Ceballos Rasgado (M.C.R.), PhD in Public Health, Msc in Nutrition Physical Activity and Public Health. BSc in Nutrition and Food Science. A mixed-methods researcher with more than 5 years’ experience in research in the area of Public Health Nutrition. Mexican National, living in the UK, at the University of Central Lancashire. Research interests: Mother and child nutrition; middle-class woman on her 30’s.

MPH Pedro Javier Mota-Castillo (P.M.C), master’s in public health, Specialist in Food’s Anthropology, Nutritionist. More than 5 years’ experience in research in qualitative and social research on breastfeeding and Public Health. Mexican National. Research interests in qualitative and social research for improve health’s and food’s politics; middle-class man on his 30’s.

MPH Andrea Santos-Guzman (A.S.G.), master’s in public health, 4 years of experience in research in Public Health Nutrition. She worked at the National Institute of Public Health while she was involved in this project. Currently residing in USA.

MPH Valeria Aureoles-García (V.A.G.), master’s in public health 2 years of experience in research in public Health nutrition, currently working in the breastfeeding department of healthcare subsystem at the National Institute of Public Health Mexico.

Dr. Victoria Moran (V.L.M) has a PhD in women’s health and over 20 years of experience in conducting research in maternal and child nutrition and health. She is editor of the Maternal & Child Nutrition journal that is committed to disseminating high quality research on nutrition. VHM was not involved in the peer review or editorial decision-making process for this article.

Matthias Sachse Aguilera (M.S.), is a medical doctor, with a Master in “Public Health and Health Systems Management”. With more than 24 years of experience working in international organizations, collaborated in various research studies and coordinated multiple projects focused on improving the quality of maternal and child care. Currently working in UNICEF dedicated to the promotion of good health and nutrition for infants and young children and combating all forms of malnutrition.

Dr . Katherine Markwell (K.M.) has a PhD in Behavioural Nutrition and 11 years research experience in public health nutrition. During her participation in this project she worked at the University of Central Lancashire teaching public health nutrition and was course leader of MSc (pre-registration) in Dietetics. She currently works as Lecturer in Nutritional Behaviour and Health Promotion at the University of Leeds.

**Interviewer Facilitator:**

The interviews and focus groups were conducted and lead by P.M.C. An experienced male research assistant working for the National Institute of Public Health. During the focus groups, another team member: (A.S.G., V.A.G. and M.U.M.) were present to observe and ensure comprehensive coverage of the topic guide and facilitate deeper exploration of certain topics during interviews.

Was a relationship established prior to the study commencement?

MPH P.M.C., No prior relationships were established with the participants by this researcher before the commencement of the study.

MPH A.S.G., No prior relationships with the participants were established before the study began.

MPH V.A.G., No prior relationships with the participants were established before the study began.

Dr. M.U.M., No prior relationships with the participants were established before the study began.

Participant Knowledge of the Interviewers/Focus groups:

Participants were informed about the purpose of the study and reason why they have been invited to participate and how their data was going to be used. The were also informed of the institutions (University of Central Lancashire, National Institute of Public Health of Mexico) involved in this project. The participants were specifically briefed on the relevant experience and roles of P.M.C., M.U.M., V.A.G., A.S.G., and M.C.R.

How was reflexivity addressed?

All researchers engaged in ongoing self-reflection to remain aware of their own potential biases and how each of our personal backgrounds and experiences might have influenced the research process. Also, researchers involved in data analysis and collection, regularly communicated with each other to share their views and reflect on how their backgrounds and experiences might influence the research process and outcomes.

The research team has a diverse background and expertise allowing a rich and multi-faceted understanding of the data. The iterative approach to data analysis, involved multiple rounds of coding and agreement exercises. This helped us to identify and mitigate any biases to ensure that the final themes in this report are grounded in the data that was collected.
