# Supplementary material for: Formative Research for the Development and Implementation of a Smartphone Application to Report Breaches to the International Code of Marketing of Breast‐Milk Substitutes in Mexico
Source: Matern Child Nutr. 2025 Mar 18;21(3):e70014. doi: 10.1111/mcn.70014 (PMC12150152; doi:10.1111/mcn.70014)

Contents

[Topic Guides 2](#_Toc172547429)

[Questionnaire 20](#_Toc172547430)

[Invitation 22](#_Toc172547431)

# Topic Guides


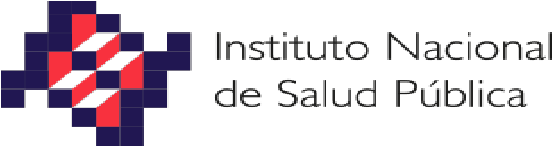


Focus Group Topic Guide for Decision Makers (Government)

**General data of the interview (only mention them on the recorder):**

- Interview date
- Interview Number
- State

Greetings, my name is _______ and my colleague’s name is ______. We are staff from the National Institute of Public Health (INSP), and are currently working in a research project aiming to generate new knowledge to inform the design and usability of a new public health surveillance app to monitor breast milk substitutes breaches to The International Code of Marketing of Breast milk Substitutes (the Code) (the Code) and explore the specific barriers and facilitators influencing stakeholders’ use of the app.

As we explained to you when the invitation was made, this conversation is completely anonymous and confidential. What we talk about here will not be discussed with anyone not directly participating in the project. None of the reports resulted from this project will have your name or any information that could be associated to you. It is important to remind you that you have the right not to answer any questions and that you can terminate your participation within the group at any time you wish without being forced to give an explanation. The focus group discussion will have a length for approximate 60-90 minutes.

The focus group discussion will be recorded to enable to analysis of the information discussed today. Recordings collected today will be password protected, and only the research team will have access to these. During data analysis names will not be used, but a code (identifier). Once the study is finished, recordings will be safeguarded for a period of five years and then safely destroyed.

Can each of you please let me know if you a) Have any questions? b) Agree with the focus group discussion being recorded?

If participants agree to be enrolled and focus groups recorded, Ask participants to mention their name and the phrase "I understand what has been read to me and I agree to participate and that this focus group discussion is recorded "

In the consent form I gave you can find information of those responsible for the study and the ethics committee of the INSP, in case you have any questions related to the study and to the information you provide after the group. So far, do you have any questions?

Before we begin, I would like to propose some rules, you can propose some others for a better development of the group

• Discretion and trustworthy environment

• All ideas and opinions are valid and there are no right or wrong answers to the questions asked during the focus groups.

• Proposals to spend a pleasant, entertaining, and dynamic time are welcome.

• Anyone can leave the activity at any time they want.

**1.- Introduction**

To begin with, I would like to ask you some personal information.

- Could you tell me, how old are you? What is your profession?

**2 Awareness and/or views of traditional (tv, radio, newspaper, magazines) and digital marketing on people’s decisions to purchase breast milk substitutes, commercial milk formula and baby foods.**

- Have you seen any marketing of any marketing of breast milk substitutes, commercial milk formula and baby foods? Where have you seen this? What are your thoughts about it?

**3 Awareness, views, beliefs, attitudes and understanding of the International Code of Breast Milk Substitutes.**

- Have you heard of the International Code of Breast milk Substitutes? If so, where do you heard of it?

*Briefly explain the Code for those not familiar with it:*

The International Code of Marketing of Breast milk Substitutes (the Code) is an international health policy framework to regulate the marketing of breast milk substitutes to protect breastfeeding. The companies of infant formulas; follow on formulas; infant milks marketed as food for special medical purposes (FSMP); baby foods; bottles/teats and related equipment may not: promote their products in hospitals, shops or to the general public; give free samples to mothers or free or subsidised supplies to hospitals or maternity wards; give gifts to health workers or mothers; promote their products to health workers: any information provided by companies must contain only scientific and factual information; promote foods or drinks for babies; give misleading information; have direct contact with mothers.

- What do you think about it (e.g. is it important?)
- Have you ever witnessed a violation to this Code?
- Do you think that the Code is relevant to you? Why?
- Who do you think should monitor compliance with the Code and sanction non-compliers in Mexico? Why do you think so?
- What do you think that the consequences should be for non-compliance?
- If you saw any violations to this code, would you report any breaches to it (why)?
- What would make you more likely to be interested in reporting breaches to the code? (e.g. something related to the form or promoting the product (e.g. a health professional, institution or online ad)? something related to the process to follow to report it? the impact that reporting could do?)
- What would make you less likely to be interested in reporting breaches to the code? (e.g. something related to the form or promoting the product who are where you saw it advertised (e.g. a health professional, institution or online ad)? something related to the process to follow to report it? the impact that reporting could do?)
- How should a monitoring system be structured to improve compliance with the Code and sanction non-compliance in Mexico?
- What do you think the consequences should be for companies and professionals who break the code?
- Have you ever wanted or attempted to report or raise a complaint a breach of any law or something you perceived was not right? For example, a breach to the Code or a law (For example: a business selling tobacco or alcohol to minors?) If so, why did you do it? if not why you did not do it?
- What do you think would be the ideal channel to report breaches of the code?

**4. Mobile app usage, and positive and negative experiences using mobile apps**

- How well do they usually handle electronic devices, for example, tablets or smartphones? How often do they use these devices? How easy or difficult is it for you to download apps?
- Do you often ask for help installing or updating an application? From who or whom? How easy or difficult is it for you to use an app? Which ones do you use most often?
- Do you know any application any information on breastfeeding or infant feeding? If so, what do you like about it and what you don’t like about it?

1. **Knowledge, Attitudes and Practices towards a new surveillance tool to monitor for breast milk substitutes, specifically commercial milk formula and baby food breaches to the Code.**

We are planning to design a digital application where people could report breaches of the Code and provide evidence such as photographs or screenshots, as well as record the place where the add was seen, or report inappropriate industry practices; for example, incentives offered to health professionals to promote their products, industry sponsored health conferences.

- Do you think that about the idea of the development of this app? Do you think it would be helpful to protect breastfeeding and young child’s nutrition?
- What would make you likely to **download** the app? Why?
- What barriers would you have to **download** the app? Why? What could we do to reduce those barriers?
- What things would make them uninstall immediately? What could we do to reduce those barriers?
- What content you think this app should have to make it **useful to you**? How does this information should be presented to make it easier to understand and more user-friendly? What barriers you think would make you unlikely to **use** the app? What could we do to reduce those barriers?
- What content you think this app should have to make it useful to the parents and would make you recommend it among them?
- If you had the app, what would make you more likely to report a breach to the code? What would make you less likely to not report a breach to the code?
- If you had the app, what kind of things would you report? If you had the app what things you would not report?
- Do you think medical staff breaching the code should be reported? Would you report medical staff or just the publicity or company?

In relation to the process of reporting a breach to the code:

- How does the instructions of the process to report a breach should be presented to make it easier to understand and more user-friendly? Do you think a video tutorial would be useful to learn how to use it/how to make complaints? Would you like the app to notify you with a follow up on your report?
- How many steps or time spent filling a complaint would you consider to be (Fair?) to make the complaint? Would you suggest another option besides the ones we already mentioned?
- Would you be able to take photographs or screenshots to report a breach to the code?
- What other functions do you think that this app should have? What would you like the app to notify you?

In terms of the processing of the information:

- Who do you think should be in charge of processing the violations to the code obtained from the app?
- What considerations do you think we would need to take into account in terms of data protection? for example to protect the identity of doctors and hospitals that are reported

Promoting the app:

- How and where and how do you think this app should be promoted?

We have reached the end of this exercise, is there anything else you would like to add, did we forgot to ask you something that you consider is important?

Thank you for your time and participation.

The recording stops.


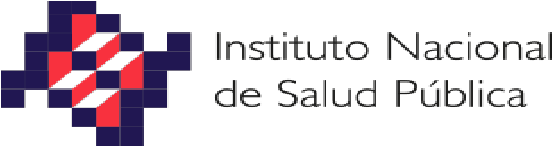


**A Qualitative Study of Mexican Stakeholders to Inform the Design and Usability of a new Smartphone Application to Report Breaches to the Marketing CODE of Formula Milk and Infant Foods Advertising in Mexico**

Focus Group Topic Guide for Health professionals

**General data of the interview (only mention them on the recorder):**

- Interview date:_____
- Focus group Number:_______

Greetings, my name is _______ and my colleague’s name is ______. We are staff from the National Institute of Public Health (INSP). We are currently working in a research project that aims to generate new knowledge to inform the design and usability of a new public health surveillance app to monitor breaches to The International Code of Marketing of Breastmilk Substitutes (the Code) and explore the specific barriers and facilitators influencing stakeholders’ use of the app.

The conversation about to be held is completely anonymous and confidential. What we talk about here will not be discussed with anyone not directly participating in the project. None of the reports resulted from this project will have your name or any information that could be associated to you.

You have the right not to answer any questions, and you can terminate your participation within the group at any time you wish, without being forced to give an explanation. The focus group discussion will have an approximate length of 60-90 minutes.

The focus group discussion will be recorded to enable the analysis of the information discussed today. Recordings collected today will be password protected, and only the research team will have access to these. During data analysis, names will not be used, but a code (identifier). At the end of the study, recordings will be safeguarded for five years, and then safely destroyed.

Can each of you please let me know if you a) Have any questions? b) Agree with the focus group discussion being recorded?

If participants agree to be enrolled and focus groups recorded, ask participants to mention their name and the phrase "I understand what has been read to me, and I agree to participate and that this focus group discussion is recorded".

In the consent form provided to you, you can find information of those responsible for the study and contact details of the INSP ethics committee of the INSP in case you have any questions related to the study, and the information you provide after the group. So far, do you have any questions?

Before we begin, I would like to propose some rules. You can propose some others for a better development of the group

• Discretion, trustworthy and respectful environment

• All ideas and opinions are valid and there are no right or wrong answers to the questions asked during the focus groups.

• Suggestions to spend a pleasant, entertaining, and dynamic time are welcome.

**1 Introduction**

To begin with, can you please introduce yourself? Tell us where do you work and what do you do?

**2 Awareness and/or views of traditional (tv, radio, newspaper, magazines) and digital marketing on people’s decisions to purchase** **breastmilk substitutes, commercial milk formula and baby foods.**

- Have you seen any marketing of any marketing of breastmilk substitutes, commercial milk formula and baby foods? Where have you seen this? What are your thoughts about it?
- Have you ever been given any branded merchandise with logo of any breastmilk substitutes, commercial milk formula and baby foods to keep in your clinic? What do you think of these? Do you keep them? Why?
- Have you ever been given free samples of breastmilk substitutes, commercial milk formula and baby foods? Did you give these to your patients? Why did you promote these or not?
- Are there any breastmilk substitutes, commercial milk formula and baby foods that you promote with your patients? What do you is it and why you promote it?

**3 Awareness, views, beliefs, attitudes and understanding of the International Code of Breast Milk Substitutes.**

- Have you heard of the International Code of Breastmilk Substitutes? If so, where do you heard of it?

*Briefly explain the Code for those not familiar with it:*

The International Code of Marketing of Breastmilk Substitutes (the Code) is an international health policy framework to regulate the marketing of breastmilk substitutes to protect breastfeeding. The companies of infant formulas; follow on formulas; infant milks marketed as food for special medical purposes (FSMP); baby foods; bottles/teats and related equipment may not: promote their products in hospitals, shops or to the general public; give free samples to mothers or free or subsidised supplies to hospitals or maternity wards; give gifts to health workers or mothers; promote their products to health workers: any information provided by companies must contain only scientific and factual information; promote foods or drinks for babies; give misleading information; have direct contact with mothers.

- What do you think about it (e.g. is it important?)
- Do you think that the Code is relevant to you? Why?
- Have you ever witnessed a violation in your working place? Can you tell us what happened?
- If you saw any violations to this code, would you report any breaches to it (why)?
- What would make you more likely to be interested in reporting breaches to the code? (e.g. something related to the form or promoting the product (e.g. a health professional, institution or online ad)? something related to the process to follow to report it? the impact that reporting could do?)
- What would make you less likely to be interested in reporting breaches to the code? (e.g. something related to the form or promoting the product who are where you saw it advertised (e.g. a health professional, institution or online ad)? something related to the process to follow to report it? the impact that reporting could do?)
- Who do you think should monitor compliance with the Code and sanction non-compliers in Mexico?
- Have you ever wanted or attempted to report or raise a complaint or a breach of any law or something you perceived was not right? For example, a breach to the Code or a law (For example: a business selling tobacco or alcohol to minors?) If so, why did you do it? if not why you did not do it?
- Do you think there should be consequences when companies do not follow the Code? Do you think there should be consequences when health professionals do not follow the Code? What do you think these should be?

**4. Mobile app usage, and positive and negative experiences using mobile apps**

- How well do they usually handle electronic devices, for example, tablets or smartphones? How often do they use these devices? How easy or difficult is it for you to download apps?
- Do you often ask for help installing or updating an application? From who or whom? How easy or difficult is it for you to use an app? Which ones do you use most often?
- Do you use apps in your daily clinical practice? Which one do you use? What do they like about these apps?
- Do you recommend any apps to your patients? Why do you recommend these? Does this apps have any information on breastfeeding or infant feeding?
- Do you know any application any information on breastfeeding or infant feeding? If so, what do you like about it and what you don’t like about it?

1. **Knowledge, Attitudes and Practices towards a new surveillance tool to monitor for breastmilk substitutes, specifically commercial milk formula and baby food breaches to the Code.**

We are planning to design a digital application where people could report breaches of the Code and provide evidence such as photographs or screenshots, as well as record the place where the add was seen, or report inappropriate industry practices; for example, incentives offered to health professionals to promote their products, industry sponsored health conferences.

- Do you think that about the idea of the development of this app? Do you think it would be helpful to protect breastfeeding and young child’s nutrition?
- What would make you likely to **download** the app? Why?
- What barriers would you have to **download** the app? Why? What could we do to reduce those barriers?
- What things would make them uninstall immediately? What could we do to reduce those barriers?
- What content you think this app should have to make it useful to you? How does this information should be presented to make it easier to understand and more user-friendly? What barriers you think would make you unlikely to **use** the app? What could we do to reduce those barriers?
- What content you think this app should have to make it useful to the parents and would make you recommend it among them?
- If you had the app, what would make you more likely to report a breach to the code? What would make you less likely to not report a breach to the code (e.g. features of the app, consequences of the report)?
- If you had the app, what kind of things would you report? If you had the app what things, you would not report? (e.g. report adds but not colleague giving away free samples?)
- Do you think medical staff breaching the code should be reported? Would you report medical staff or just the publicity or company?
- How does the instructions of the process to report a breach should be presented to make it easier to understand and more user-friendly? Do you think a video tutorial would be useful to learn how to use it/how to make complaints? Would you like the app to notify you with a follow up on your report?
- What is the time you would consider is accepable to spend reporting a breach to the Code?
- Would you be able to take photographs or screenshots to report a breach to the code?
- Who do you think should be in charge of processing the violations to the code obtained from the app?
- What other functions do you think that this app should have? What would you like the app to notify you?

Promoting the app:

- How and where and how do you think this app should be promoted?

We have reached the end of this exercise, is there anything else you would like to add, did we forgot to ask you something that you consider is important?

Thank you for your time and participation.

The recording stops.


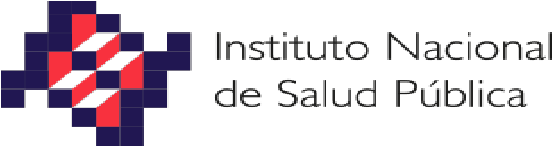


**A Qualitative Study of Mexican Stakeholders to Inform the Design and Usability of a new Smartphone Application to Report Breaches to the Marketing CODE of Formula Milk and Infant Foods Advertising in Mexico**

Focus Group Topic Guide for mothers, fathers and caregivers of young children

**General data of the interview (only mention them on the recorder):**

- Interview date:_____
- Focus group Number:_______

Greetings, my name is _______ and my colleague’s name is ______. We are staff from the National Institute of Public Health (INSP). We are currently working in a research project that aims to generate new knowledge to inform the design and usability of a new public health surveillance app to monitor breaches to The International Code of Marketing of Breastmilk Substitutes (the Code) and explore the specific barriers and facilitators influencing stakeholders’ use of the app.

The conversation about to be held is completely anonymous and confidential. What we talk about here will not be discussed with anyone not directly participating in the project. None of the reports resulted from this project will have your name or any information that could be associated to you.

You have the right not to answer any questions, and you can terminate your participation within the group at any time you wish, without being forced to give an explanation. The focus group discussion will have an approximate length of 60-90 minutes.

The focus group discussion will be recorded to enable the analysis of the information discussed today. Recordings collected today will be password protected, and only the research team will have access to these. During data analysis, names will not be used, but a code (identifier). At the end of the study, recordings will be safeguarded for five years, and then safely destroyed.

Can each of you please let me know if you a) Have any questions? b) Agree with the focus group discussion being recorded?

If participants agree to be enrolled and focus groups recorded, ask participants to mention their name and the phrase "I understand what has been read to me, and I agree to participate and that this focus group discussion is recorded".

In the consent form provided to you, you can find information of those responsible for the study and contact details of the INSP ethics committee of the INSP in case you have any questions related to the study, and the information you provide after the group. So far, do you have any questions?

Before we begin, I would like to propose some rules. You can propose some others for a better development of the group

• Discretion, trustworthy and respectful environment

• All ideas and opinions are valid, and there is no right or wrong answer to the questions that will be asked in this focus group.

• Suggestions to spend a pleasant, entertaining, and dynamic time are welcome.

**1. Introduction**

- To begin with, could you please introduce yourself and tell us, how old are your children or the children you care?

**2. Awareness and/or views of traditional (tv, radio, newspaper, magazines) and digital marketing on people’s decisions to purchase breastmilk substitutes, commercial milk formula and baby foods.**

- Have you ever seen marketing of breastmilk substitutes, commercial milk formula and baby foods? If so, what was it? where was it? what tools or strategies were being used to advertise it (e.g., what media imagery, discount coupons, banners etc.)? What did you think when you saw it? Do you think that the ad you saw could influence other people´s decision to buy the product? Why do you think so?
- Have you seen any advertising in health institutions? Did any health professional ever recommended you using formula instead of breastfeeding? If so, why? What brand?

**3 Awareness, views, beliefs, attitudes and understanding of the International Code of Breast Milk Substitutes.**

- Have you heard of the International Code of Breastmilk Substitutes?

*Briefly explain the Code for those not familiar with it.*

The International Code of Marketing of Breastmilk Substitutes (the Code) is an international health policy framework to regulate the marketing of breastmilk substitutes in order to protect breastfeeding. The companies of infant formulas; follow on formulas; infant milks marketed as food for special medical purposes (FSMP); baby foods; bottles/teats and related equipment may not: promote their products in hospitals, shops or to the general public; give free samples to mothers or free or subsidised supplies to hospitals or maternity wards; give gifts to health workers or mothers; promote their products to health workers: any information provided by companies must contain only scientific and factual information; promote foods or drinks for babies; give misleading information; have direct contact with mothers.

- What do you think about it (e.g. is it important?)
- Do you think that the Code is relevant to you? Why?
- If you saw any violations to this code would you report any breaches to it (why)?
- What would make you more likely to be interested in reporting breaches to the code? (e.g. something related to an ad you saw or the channel advertising it (e.g. a health professional, institution or online ad)? something related to the process to follow to report it?, the impact that reporting could do?)
- What would make you less likely to be interested in reporting breaches to the code? (e.g. something related to who or where you saw it advertised (e.g. a health professional, institution or online ad)? something related to the process to follow to report it? the impact that reporting could do?)
- Who do you think should monitor compliance with the Code and sanction non-compliers in Mexico?
- Have you ever wanted or attempted to report or raise a complaint a breach of any law or something you perceived was not right? For example, a breach to the Code or a law (For example: a business selling tobacco or alcohol to minors?) If so, why did you do it? if not why you did not do it?

4. **Mobile app usage, and positive and negative experiences using mobile apps**

- How well do they usually handle electronic devices, for example, tablets or smartphones? How often do they use these devices? How easy or difficult is it for you to download apps?
- Do you often ask for help installing or updating an application? From who or whom? How easy or difficult is it for you to use an app? Which ones do you use most often?
- Is there any parenting related app that you use? What do you like about it? Who recommended it to you? Does it has information about infant and young child feeding?
- Have you ever downloaded an app that has provided you with information around breastfeeding and/or complementary feeding or young child’s feeding? How often do you use it? Is there anything on it that you found useful (what)? Is there something that you like about it? Is there something that you don’t like about it? Who recommended it to you or how did you know about it? Have you ever recommended any app to a member of your family or friend? Why did you do it?
- Have you use any brand sponsored apps that provide information around breastfeeding and/or complementary feeding or young child’s feeding? What do you think of it?
- Has there been an app related to infant and young child feeding that you downloaded that you never used or did not like? Why did you not like/use it?
- For those that have never downloaded any app related to young child nutrition, why have you never done it? Would you do it if they had one recommended to them? What would it need to have to be useful to them?

5. **Knowledge, Attitudes and Practices towards a new surveillance tool to monitor for breastmilk substitutes, specifically commercial milk formula and baby food breaches to the Code.**

We are planning to design a digital application where people could report breaches of the Code and provide evidence such as photographs or screenshots, as well as record the place where the add was seen, or report inappropriate industry practices; for example, incentives offered to health professionals to promote their products, industry sponsored health conferences.

- What would make you likely to download the app? Why?
- What barriers would you have to **download** the app? Why?
- What content you think this app should have to make it useful to you? How does this information should be presented to make it easier to understand and more user-friendly? What barriers you think would make you unlikely to **use** the app? What could we do to reduce those barriers?
- What things would make them uninstall immediately? What could we do to reduce those barriers?
- What things would make you keep the app?
- If you had the app, what would make you more likely to report a breach to the code? What would make you less likely to not report a breach to the code? Do you think medical staff breaching the code should be reported? Would you report medical staff or just the publicity?
- How does the instructions of the process to report a breach should be presented to make it easier to understand and more user-friendly? Do you think a video tutorial would be useful to learn how to use it/how to make complaints? Would you like the app to notify you with a follow up on your report?
- What is the time you would consider is acceptable to spend reporting a breach to the Code?
- Would you be able to take photographs or screenshots to report a breach to the code?
- What other functions do you think that this app should have? What would you like the app to notify you?
- Where and how do you think this app should be promoted?

We have reached the end of this exercise, is there anything else you would like to add, did we forgot to ask you something that you consider is important?

Thank you for your time and participation.

The recording stops.


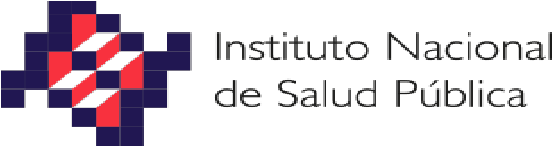


**A Qualitative Study of Mexican Stakeholders to Inform the Design and Usability of a new Smartphone Application to Report Breaches to the Marketing CODE of Formula Milk and Infant Foods Advertising in Mexico**

Focus Group Topic Guide for stakeholders

General data of the interview (only mention them on the recorder):

- Interview date:_____
- Focus group Number:_______

Greetings, my name is _______ and my colleague’s name is ______. We are staff from the National Institute of Public Health (INSP). We are currently working in a research project that aims to generate new knowledge to inform the design and usability of a new public health surveillance app to monitor breaches to The International Code of Marketing of Breastmilk Substitutes (the Code) and explore the specific barriers and facilitators influencing stakeholders’ use of the app.

The conversation about to be held is completely anonymous and confidential. What we talk about here will not be discussed with anyone not directly participating in the project. None of the reports resulted from this project will have your name or any information that could be associated to you.

You have the right not to answer any questions, and you can terminate your participation within the group at any time you wish, without being forced to give an explanation. The focus group discussion will have an approximate length of 60-90 minutes.

The focus group discussion will be recorded to enable the analysis of the information discussed today. Recordings collected today will be password protected, and only the research team will have access to these. During data analysis, names will not be used, but a code (identifier). At the end of the study, recordings will be safeguarded for five years, and then safely destroyed.

Can each of you please let me know if you a) Have any questions? b) Agree with the focus group discussion being recorded?

If participants agree to be enrolled and focus groups recorded, ask participants to mention their name and the phrase "I understand what has been read to me, and I agree to participate and that this focus group discussion is recorded".

In the consent form provided to you, you can find information of those responsible for the study and contact details of the INSP ethics committee of the INSP in case you have any questions related to the study, and the information you provide after the group. So far, do you have any questions?

Before we begin, I would like to propose some rules. You can propose some others for a better development of the group

• Discretion, trustworthy and respectful environment

• All ideas and opinions are valid, and there is no right or wrong answer to the questions that will be asked in this focus group.

• Suggestions to spend a pleasant, entertaining, and dynamic time are welcome.

1. **Introduction**

To begin with, could you please introduce yourself? Where do you work? What type of work do you do in your organization?

**3.- Knowledge**

**2 Awareness and/or views of traditional (tv, radio, newspaper, magazines) and digital marketing on people’s decisions to purchase breastmilk substitutes, commercial milk formula and baby foods.**

- Have you seen any marketing of any marketing of breastmilk substitutes, commercial milk formula and baby foods? Where have you seen this? What are your thoughts about it?

**3 Awareness, views, beliefs, attitudes and understanding of the International Code of Breast Milk Substitutes.**

- Have you heard of the International Code of Breastmilk Substitutes? If so, where do you heard of it?

*Briefly explain the Code for those not familiar with it:*

The International Code of Marketing of Breastmilk Substitutes (the Code) is an international health policy framework to regulate the marketing of breastmilk substitutes to protect breastfeeding. The companies of infant formulas; follow on formulas; infant milks marketed as food for special medical purposes (FSMP); baby foods; bottles/teats and related equipment may not: promote their products in hospitals, shops or to the general public; give free samples to mothers or free or subsidised supplies to hospitals or maternity wards; give gifts to health workers or mothers; promote their products to health workers: any information provided by companies must contain only scientific and factual information; promote foods or drinks for babies; give misleading information; have direct contact with mothers.

- What do you think about it (e.g. is it important?)
- Have you ever witnessed a violation to this Code?
- Do you think that the Code is relevant to you? Why?
- Who do you think should monitor compliance with the Code and sanction non-compliers in Mexico? Why do you think so?
- What do you think that the consequences should be for non-compliance?
- If you saw any violations to this code, would you report any breaches to it (why)?
- What would make you more likely to be interested in reporting breaches to the code? (e.g. something related to the form or promoting the product (e.g. a health professional, institution or online ad)? something related to the process to follow to report it? the impact that reporting could do?)
- What would make you less likely to be interested in reporting breaches to the code? (e.g. something related to the form or promoting the product who are where you saw it advertised (e.g. a health professional, institution or online ad)? something related to the process to follow to report it? the impact that reporting could do?)
- How should a monitoring system be structured to improve compliance with the Code and sanction non-compliance in Mexico?
- What do you think the consequences should be for companies and professionals who break the code?

**4. Mobile app usage, and positive and negative experiences using mobile apps**

- How well do they usually handle electronic devices, for example, tablets or smartphones? How often do they use these devices? How easy or difficult is it for you to download apps?
- Do you often ask for help installing or updating an application? From who or whom? How easy or difficult is it for you to use an app? Which ones do you use most often?
- Do you know any application any information on breastfeeding or infant feeding? If so, what do you like about it and what you don’t like about it?

1. **Knowledge, Attitudes and Practices towards a new surveillance tool to monitor for breastmilk substitutes, specifically commercial milk formula and baby food breaches to the Code.**

We are planning to design a digital application where people could report breaches of the Code and provide evidence such as photographs or screenshots, as well as record the place where the add was seen, or report inappropriate industry practices; for example, incentives offered to health professionals to promote their products, industry sponsored health conferences.

- Do you think that about the idea of the development of this app? Do you think it would be helpful to protect breastfeeding and young child’s nutrition?
- What would make you likely to **download** the app? Why?
- What barriers would you have to **download** the app? Why? What could we do to reduce those barriers?
- What things would make them uninstall immediately? What could we do to reduce those barriers?
- What content you think this app should have to make it **useful to you**? How does this information should be presented to make it easier to understand and more user-friendly? What barriers you think would make you unlikely to **use** the app? What could we do to reduce those barriers?
- What content you think this app should have to make it useful to the parents and would make you recommend it among them?
- If you had the app, what would make you more likely to report a breach to the code? What would make you less likely to not report a breach to the code? What elements would help the complaint and what elements would hinder it_
- If you had the app, what kind of things would you report? If you had the app what things, you would not report?
- Do you think medical staff breaching the code should be reported? Would you report medical staff or just the publicity or company?

In relation to the process of reporting a breach to the code:

- How does the instructions of the process to report a breach should be presented to make it easier to understand and more user-friendly? Do you think a video tutorial would be useful to learn how to use it/how to make complaints? Would you like the app to notify you with a follow up on your report?
- What is the time you would consider is accepable to spend reporting a breach to the Code?
- Would you be able to take photographs or screenshots to report a breach to the code?
- What other functions do you think that this app should have? What would you like the app to notify you?

In terms of the processing of the information:

- Who do you think should be in charge of processing the violations to the code obtained from the app?
- What considerations do you think we would need to take into account in terms of data protection? for example, to protect the identity of doctors and hospitals that are reported

Promoting the app:

- How and where and how do you think this app should be promoted?

We have reached the end of this exercise, is there anything else you would like to add, did we forgot to ask you something that you consider is important?

Thank you for your time and participation.

The recording stops.


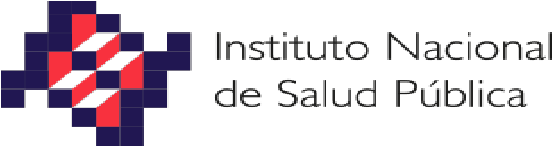


**A Qualitative Study of Mexican Stakeholders to Inform the Design and Usability of a new Smartphone Application to Report Breaches to the Marketing CODE of Formula Milk and Infant Foods Advertising in Mexico**

Questionnaire for health professionals:

- Gender: Male____ Female_____
- How old are you? ________ years.
- How long have you been working in the health sector: ____months ______ years.
- What is your profession? __________
- Do you give consultation? Yes_____ No____
- Where do you give consultation? _______ Private sector. ________Public sector.
- How long have you been working in this institution:

Public:_______ years, ________ months

Private:_______ years, ________ months

- Are you part of a professional association? Yes_____ No____

# Questionnaire

______


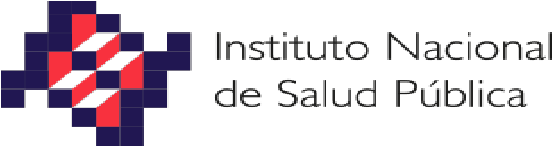
**A Qualitative Study of Mexican Stakeholders to Inform the Design and Usability of a new Smartphone Application to Report Breaches to the Marketing CODE of Formula Milk and Infant Foods Advertising in Mexico**

Questionnaire for mothers, fathers, and caregivers of young children:

- Gender: Male____ Female_____
- What is your profession? ______
- Until what year did you go to school? Did not go to school _____ Primary/secondary school_______ High school ______ Vocational_______ truncated University ______ Completed University_________
- How many children do you have? _________
- What age is your youngest child or the child you care? ____months _____ years.
- Do you have a partner/spouse? yes____ no____
- Do you live with your partner/spouse? yes____ no____


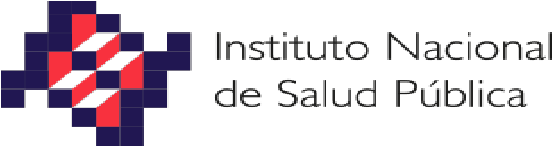


**A Qualitative Study of Mexican Stakeholders to Inform the Design and Usability of a new Smartphone Application to Report Breaches to the Marketing CODE of Formula Milk and Infant Foods Advertising in Mexico**

Questionnaire for stakeholders (NGOs and Civil Society):

- Gender: Male____ Female_____
- How old are you? ________ years.
- Where do you work? ________

______ years, ________ months

- What is your profession? __________
- How long have you been working in this institution:

______ years, ________ months

- What are the activities carried out by the organization association to which you belong?
- What are the tasks you do within the association/organization/unit/Secretariat?

# Invitations


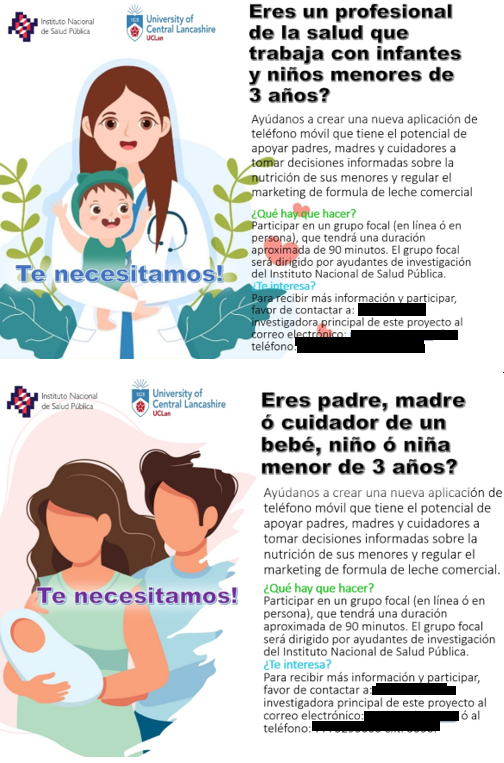

Supplement: Supplementary file 2 — Supporting information. [file MCN-21-e70014-s002.docx]
